# Ceftriaxone-Induced Reversible Agranulocytosis: A Case Report and Review of Drug-Induced Agranulocytosis

**DOI:** 10.7759/cureus.23226

**Published:** 2022-03-16

**Authors:** Farrukh Munir, Hafiza Wajeeha Javaid, Muhammad Burhan Majeed Rana, Fatima Shaukat

**Affiliations:** 1 Internal Medicine, WellSpan York Hospital, York, USA; 2 Internal Medicine, Queens Hospital Center, New York, USA; 3 Infectious Disease, University of Maryland (UM) Laurel Medical Center, Laurel, USA

**Keywords:** bone marrow biospy, neutropenia, g-csf, drug induced agranulocytosis, ceftriaxone induced aganulocytosis

## Abstract

In the modern era of medicine, agranulocytosis is a rare occurrence. Despite significant improvement in patient survival, it still carries significant mortality. Agranulocytosis is most commonly caused by chemotherapeutic agents and numerous non-chemo drugs. As it can develop anytime during treatment and patients can remain asymptomatic, frequent cell count monitoring is an essential tool to make a timely diagnosis. An appropriate drug switch, work up to rule out infection and granulocyte colony-stimulating factor (G-CSF) injection in high-risk cases is the management. The patient should be kept under observation till the resolution of agranulocytosis. We present a case of ceftriaxone-induced agranulocytosis which was completely reversible upon stoppage of drug and granulocyte colony-stimulating factor administration. The pathogenesis of ceftriaxone-induced agranulocytosis is unknown. It is suggested to occur either by an immunologic mechanism or because of direct drug toxicity.

## Introduction

Agranulocytosis is defined as an absolute neutrophil count of zero. It has a historically high mortality rate of up to 16%. Although with recent advancements the mortality has decreased to 5%, this condition is still considered life-threatening, especially in patients who have underlying bacteremia, renal failure, shock, or age greater than 65 years at the time of diagnosis [1]. Chemotherapeutic agents are the most common culprits however some commonly used non-chemo drugs like methimazole, bisoprolol, clozapine, amitriptyline, ketorolac, chloroquine, and certain beta-lactam antibiotics have also been known to cause agranulocytosis. We present a case of ceftriaxone-induced agranulocytosis which was completely resolved upon stoppage of drug and granulocyte colony-stimulating factor administration. The pathogenesis of ceftriaxone-induced agranulocytosis is unknown. It is suggested to occur either by an immunologic mechanism or because of direct drug toxicity [2].

## Case presentation

A 78-year-old Caucasian male with a history of coronary artery disease, type 2 diabetes mellitus, normocytic anemia was admitted with septic left ankle joint arthritis with Streptococcus mitis bacteremia. Vancomycin and piperacillin/tazobactam were initiated empirically, and arthroscopic I & D was performed. Transthoracic echocardiogram (TTE) and transesophageal echocardiogram (TEE) were both negative for any vegetations. His antibiotics were deescalated to ceftriaxone 2 g daily for six weeks according to culture sensitivities. He was followed up on an outpatient basis by weekly complete blood count (CBC) and differential, creatinine, erythrocyte sedimentation rate (ESR), and C-reactive protein (CRP). On day 38 of ceftriaxone, his labs showed agranulocytosis. He was afebrile and was admitted. Repeat labs showed absolute neutrophil count (ANC) of zero; he remained afebrile. Adverse drug reaction probability score (Naranjo score) was 7, which indicated probable drug reaction [3]. Ceftriaxone was stopped immediately, and vancomycin and meropenem were initiated. Filgrastim was also administered. Sepsis workup including blood cultures, urinalysis and culture, respiratory viral panel, and radiograph of the chest was normal. His agranulocytosis resolved the next day after discontinuation of ceftriaxone and administration of filgrastim. His antibiotics were switched to PO amoxicillin to complete a total six-week course and he was discharged. One-week follow-up labs showed no recurrence of agranulocytosis. Table 1 shows patient's white blood cell (WBC) count pretreatment, the drop in WBCs with ceftriaxone, and recovery in WBCs after stopping the offending agent. 

**Table 1:** Laboratory Values ANC: absolute neutrophil count; WBC: white blood cell

	WBC	ANC	Lymphocytes	Hemoglobin	Platelets
1-week pretreatment	9.9	7.99	0.85	9.7	262
Admission day	10.2	9.69	0.99	8.8	265
Day 1 of ceftriaxone	7.5	5.53	1.05	7.5	267
Day 14 of ceftriaxone	8.2	6.42	0.92	8.2	309
Day 28 of ceftriaxone	6.2	4.03	1.26	9.2	315
Day 38 of ceftriaxone, Ceftriaxone stopped, Amoxicillin started	1.7	0.00	0.75	9.0	318
Day 2 of amoxicillin, Tbo-filgrastim administered	1.4	0.02	0.60	8.0	321
1 Day after Tbo-filgrastim	5.7	2.74	1.55	7.5	286
7 Days after Tbo-filgrastim	6.8	4.04	1.35	9.0	334

## Discussion

What are neutrophils?

Neutrophils are the major defense mechanism in innate immunity against bacterial and fungal infections. They are the most abundant type of WBCs and constitute approximately 60% of all WBCs in human blood circulation. Their production in the bone marrow is regulated by cytokine granulocyte colony-stimulating factor (G-CSF). They remain functional for six to eight hours in circulation and get destroyed in the liver, spleen, and bone marrow [4].

Bone marrow has significant reserves of neutrophils. After infection or stress, cytokines are released which increase neutrophil release from bone marrow [5]. Neutrophils get attracted towards the capillary endothelium near the site of infection, the process called margination. Cytokines also signal endothelial cells to express certain receptors such as E-selectin, P-SELECTIN, GlyCAM-1, and CD-34, these receptors bind to Sialyl-LewisX and L-SELECTIN on neutrophils causing them to slow down and roll on the surface of the endothelium. The next step is the tight binding of neutrophils to the endothelium due to the binding of ICAM-1 (CD-54) and VCAM-1 (CD-106) on the endothelium to CD11/18 integrins and VLA-4 integrin on neutrophils. Once the tight bond is established, neutrophils make and extend pseudopodia between endothelial cells to leave the bloodstream and migrate to the site of infection. Both neutrophils and endothelial cells use PECAM-1 receptors for this process of diapedesis [6]. After that neutrophils follow chemotactic signals to guide their way to the site of infection [7]. After forming pseudopodia around the pathogen and engulfing it, the pathogen is killed via oxygen-dependent and lysozyme pathways [8]. Eventually, neutrophils undergo apoptosis.

Neutropenia versus agranulocytosis?

Neutropenia is an absolute neutrophil count less than 1500/microliter. Neutropenia can be mild (< 1500/microliter), moderate (< 1000/ microliter), or severe (< 500/microliter). It can result from either decreased production in bone marrow or increased destruction. Bacterial and viral infections are the most common causes of neutropenia [9]. Another category of neutropenia is benign ethnic neutropenia. It is an inherited cause of neutropenia which has more prevalence in people of African descent. Interestingly it poses no increased risk of infection [10]. 

Agranulocytosis means ANC of zero, although the term sometimes is also used for a profound degree of neutropenia i.e ANC < 100/microliter. Most cases of agranulocytosis are drug-induced and the most common causative agents are chemotherapy drugs. Other than chemotherapeutics, the following drugs mentioned in Table 2 are also reported to cause agranulocytosis.

**Table 2:** Classes of drugs that can cause neutropenia/agranulocytosis.

Drug Class	Examples	
Antithyroid drugs [11]	Methimazole, Propylthiouracil	
Antibiotics drugs [12,13]	Cephalosporins, Macrolides, TMP-SMX, Chloramphenicol, Sulfonamides, Vancomycin, Dapsone	
Anti-fungal drugs [14]	Amphotericin B, flucytosine, fluconazole	
Anti-viral drugs [15]	Oseltamivir, ganciclovir, acyclovir	
Cardiovascular drugs [16]	Procainamide, ticlopidine, captopril, propranolol, digoxin, dipyridamole, thiazides, acetazolamide, furosemide, spironolactone	
Gastrointestinal drugs [17]	Sulfasalazine, H2 receptor antagonist	
Anti-inflammatory drugs [18]	NSAIDs, penicillamine, gold salts	
Anti-convulsants [19]	Carbamazepine, phenytoin, ethosuximide, valproic acid	
Anti-psychotics [20]	TCAs, clozapine	
Miscellaneous [21,22]	Certain herbal supplements, heroin, and cocaine contaminated with Levamisole	

Epidemiology

Agranulocytosis is a rare occurrence with the incidence of 1.1 to 4.9 cases per million cases per year [23]. Agranulocytosis had a mortality rate of up to 16% which has now improved to 5% with improved recognition and management [24]. Risk factors are advancing age, female sex, infectious mononucleosis, impaired drug excretion, and underlying autoimmune disease. Poor prognostic factors include ANC < 100, age > 65, concomitant severe infection or shock, and pre-existing comorbidities like renal failure [25].

Proposed mechanisms

Both immunologic and non-immunologic mechanisms of drug-induced agranulocytosis have been proposed, but none of the mechanisms have been proven. Further research is needed in this aspect.

For immunologic mechanisms, in some cases, antibodies against neutrophils have been detected independent of the presence of the drug. It has been proposed that these culprit drugs induce autoantibody production against neutrophils [26]. Another unproven immune mechanism is the binding of immune complexes to Fc-receptors on the surface of the neutrophils. Haptens are small molecular compounds that are unable to generate an immune response on their own. They join with proteins from the body to create a hapten-protein complex that generates an immune response. Small drug molecules join with endogenous body proteins and subsequent drug-protein molecule leads to immune response. This bond between haptens and proteins can be reversible or irreversible. Some drugs require bioactivation in the body before attaching to these proteins [27].

Among non-immunologic categories, the following mechanisms have been reported. One is oxidation; drugs undergo biotransformation to produce active metabolites. Drug oxidation by myeloperoxidase leads to free radical metabolite formation leading to neutrophil destruction [28]. Genetic polymorphisms in metabolic enzymes also play a role. Human leukocyte antigen genes associated with drug-induced agranulocytosis have been studied. For example, HLA-DQB1, HLA-B was reported to increase susceptibility to clozapine-induced agranulocytosis [29]. HLA-B27 genes have also been associated with an increased risk of agranulocytosis [30].

Clinical presentation

Patients with neutropenia and agranulocytosis can be asymptomatic or present with variable clinical features including but not limited to fever, chills, generalized weakness, infections including sore throat, tonsillitis, flu-like symptoms, pneumonia, sepsis, altered mental status, and septic shock [31]. Most cases appear during the first three to six months of starting a new drug, therefore routine monitoring with CBC is recommended [32,33]. In the case of our patient, his agranulocytosis was diagnosed on outpatient labs being done for monitoring, otherwise, he remained asymptomatic. 

Diagnosis

Most patients with agranulocytosis are asymptomatic or have mild symptoms, therefore agranulocytosis is diagnosed most of the time on routine monitoring of CBC with differential. There is no specific lab test to diagnose drug-induced agranulocytosis. Differentiating between drug-induced agranulocytosis and other causes of neutropenia is essential. A detailed history is important to inquire about their diet (to rule out nutritional deficiencies including vitamin B12, copper, and folic acid), consumption of any herbal supplements, exposure to chemicals such as DDT, and radiation exposure [21,31]. Other labs should be performed to rule out HIV, myelodysplastic syndrome, hematological malignancies, and autoimmune disorders. Bone marrow biopsy can be performed if there is no obvious clinical explanation or persistent agranulocytosis/neutropenia. Bone marrow examination can also predict recovery time. Lack of myeloid precursor cells indicates longer recovery times whereas the presence of these cells with maturation arrest indicates shorter recovery times. Patients on high-risk medications should undergo monitoring labs including differential counts [34,35]. But in the case of antibiotics particularly if the patient is undergoing prolonged treatment or prescribed high doses, blood work should be performed more frequently, we suggest at least twice every week. 

Our patient was receiving 2 grams of ceftriaxone daily, on day 38 he was diagnosed with agranulocytosis with an ANC of 0.00. As the patient remained asymptomatic and his blood work was being monitored weekly, we are unsure on what day of treatment he actually developed agranulocytosis. Our patient had extensive workup including ESR, CRP, urinalysis, respiratory viral panel, parvovirus, and blood cultures including fungal cultures and they were normal. 

Treatment

If the patient is already on medications that can cause agranulocytosis, the choice of additional medications should be made carefully to prevent cumulative side effects. If agranulocytosis develops, the first step in the treatment is to stop the suspected offending drug and start to work up to rule out other causes of agranulocytosis [36]. If the offending agent is an antibiotic, a broad-spectrum antibiotic from a different class should be initiated [37]. If recovery after discontinuing the offending drug is slow or a patient is undergoing chemotherapy or secondary infection is suspected, a granulocyte colony-stimulating factor should be administered to stimulate the bone marrow to increase the production of white blood cells [37]. With G-CSF, the duration of hematological recovery, antibiotic duration, and hospitalization days is significantly reduced [38]. Some studies show improved survival with the administration of G-CSF [26].

We recommend avoiding re-exposure to the same class of drugs [24,39]. If no other treatment options are available, then re-challenge should only be considered if clinical benefits outweigh risks. In this scenario, the patient should be closely monitored with frequent monitoring of blood counts.

## Conclusions

The most common causes of acquired agranulocytosis are drugs including chemotherapy and non-chemotherapy drugs. Agranulocytosis is a serious condition that can contribute to increased mortality. It can be asymptomatic, so frequent monitoring with CBC and differential count should be done in case of prolonged treatment of drugs known to cause agranulocytosis so that it can be recognized in a timely manner. Management includes discontinuation of the offending agent, broad-spectrum antibiotics, sepsis work up, and G-CSF administration. The patient should be kept under observation till the resolution of agranulocytosis. Re-challenge should be avoided unless benefits outweigh risks.
